# Paclitaxel-Coated Balloon Angioplasty in the Real World: Jack-of-all-Trades?

**DOI:** 10.1007/s00270-020-02731-y

**Published:** 2020-12-07

**Authors:** Ulf Teichgräber

**Affiliations:** Department of Radiology, Jena University Hospital, Friedrich-Schiller-University Jena, Am Klinikum 1, 07747 Jena, Germany

Efficacy of drug-coated balloon (DCB) angioplasty is well established for femoropopliteal lesions. However, clinical benefit differs considerably across DCB types [1]. Superiority of Passeo-18 lx DCB over standard balloon angioplasty (POBA) regarding the primary endpoint of 6-month late lumen loss and the secondary endpoint of 6-month binary restenosis had been demonstrated earlier by the BIOLUX P-I randomized controlled trial (RCT). However, the small-scale study had not been powered to compare clinical endpoints, and thus, difference in the incidence of clinically driven target lesion revascularization (TLR) between groups did not reach statistical significance (DCB 15.4% vs. POBA 41.7%, *p* = 0.06) [2]. The BIOLUX P-II RCT on below-the-knee (BTK) lesions showed similar clinical outcomes after DCB angioplasty and POBA.

In this issue of CVIR, Tepe et al. [3] present 24-month results of the real-world BIOLUX P-III registry on performance and safety of infrainguinal Passeo-18 lx DCB angioplasty in 877 participants, providing specific information on femoropopliteal lesions, long lesions (≥ 15 cm), and in-stent restenosis (ISR). Results on the BTK lesion subgroup had been presented earlier in CVIR. Overall, the study population included a considerably high proportion of participants at increased risk (femoropopliteal subgroup: 43% diabetes, 33% renal disease, 41% current smoker, 35% critical limb ischemia [CLI], 25% chronic total occlusions, 17% severe calcification, and 14% in-stent restenosis). Mean femoropopliteal lesion length was 96 ± 78 mm. Investigators predilated 89% of femoropopliteal lesions with POBA and conducted bailout stenting in 20%. At 24 months, in the femoropopliteal subgroup, primary patency was 67% (imaging cohort) and freedom from TLR 89%, with considerably worse results in the long lesion- and ISR subgroups, both including BTK lesions.

Previous RCTs reported on 24-month primary patency of 57–90%, and on freedom from TLR of 64–97% after femoropopliteal DCB angioplasty [1, 4]. However, in most cases, study populations differed considerably from the BIOLUX P-III registry, particularly regarding the share of CLI participants, and thus, direct comparison is not appropriate. However, the AcoArt I [5] trial has some similarities in the study population which may enable a vague orientation on Passeo-18 lx DCB performance. AcoArt I was characterized by comparable proportions of diabetes, CLI, and bailout stenting, but higher shares of long lesions, total occlusions, and ISR. Finally, 24-month primary patency and freedom from TLR in AcoArt I were about the same order of magnitude (65%, 87%, respectively) as in the BIOLUX P-III registry.

In the BIOLUX P-III registry, all-cause mortality in the femoropopliteal subgroup was 10%. Four percent of participants experienced major amputation. Results from the long lesion- and ISR subgroups were slightly worse. In comparison, earlier RCTs on femoropopliteal DCB angioplasty reported 24-month all-cause mortality and incidence of major amputation of 1–13% and 0–6%, respectively [1, 4]. Thus, BIOLUX P-III safety outcomes were at upper end of range, probably attributed to advanced disease and comorbidities. None of the deaths were adjudicated device or procedure related. Cox regression did not identify paclitaxel dose as predictor of mortality. However, whether differences in paclitaxel dose within the study population and total number of deaths were sufficient to assess an effect on mortality might be questioned.

In conclusion, it should be considered that authors provide detailed description of cohort and subgroup characteristics and clinical and imaging outcomes that remarkably contribute to the overall assessment of DCB angioplasty. However, by the nature of registries, the major drawback of BIOLUX P-III is the absence of a control group. Therefore, the actual treatment effect of Passeo-18 lx DCB angioplasty and specific interaction effects of subgroups remain uncertain.
